# Lenticular fungal infection caused by *Aspergillus* in a patient with traumatic corneal laceration: a case report

**DOI:** 10.1186/s12886-020-01441-w

**Published:** 2020-05-01

**Authors:** Hyun Ji Hwang, Yong Woo Lee, Kyung Min Koh, Kyu Yeon Hwang, Young A Kwon, Sang Wroul Song, Byoung Yeop Kim, Kook Young Kim

**Affiliations:** Department of Cornea and Refractive Surgery, Kim’s Eye Hospital, Myunggok Eye Research Institute, Konyang University College of Medicine, 136, Yeongshin-ro, Yeongdeungpo-gu, Seoul, South Korea

**Keywords:** Aspergillosis, Corneal laceration, Endophthalmitis, Fungal infection, Intralenticular abscess

## Abstract

**Background:**

To report a case of lenticular infection caused by *Aspergillus*, which was diagnosed 13 weeks after traumatic corneal laceration.

**Case presentation:**

A 60-year-old woman presented with traumatic corneal laceration including anterior lens capsule rupture and traumatic cataract after being hit with a chestnut in the right eye. There were multiple injuries due to tiny thorns of the chestnut, including the conjunctiva, sclera, cornea, and anterior lens capsule. But no visible foreign body was detected by slit-lamp examination. Topical corticosteroid was prescribed to resolve the conjunctival inflammation induced by the thorns of chestnut, which could have caused persistent irritation. As conjunctival injection and edema being decreased during outpatient clinical follow-up, embedded conjunctival foreign body was detected and surgically removed (1st surgery). Approximately 10 weeks after the trauma, severe inflammation of the anterior segment accompanied with hypopyon developed suddenly and at the same time embedded scleral foreign body was revealed. After removal of scleral foreign body (2nd surgery), unspecified mold species was cultured from the scleral foreign body in SDA (Sabouraud dextrose agar) plate. Suspicious corneal foreign body was removed as 3rd surgery and phacoemulsification of traumatic cataract was planned as 4th surgery. Aspergillus was finally detected from removed anterior capsule and fibrotic membrane during the operation. Fungal infection resolved successfully after administration of topical (1% voriconazole and 5% natamycin) and systemic (fluconazole) antifungal agents and phacoemulsification of traumatic cataract.

**Conclusion:**

Chestnut thorns can damage multiple ocular tissues simultaneously. Lens capsular rupture could result in fungal inoculation and lead to delayed lenticular fungal infection with complicated cataract formation. In cases of ocular trauma due to organic substances such as thorns and branches, the possibility of fungal infection should be considered.

## Background

Primary intralenticular fungal infections after trauma are uncommon. Mostly, fungal infection develops slowly, and inflammatory signs, such as iridocyclitis, fungal ball, and pupillary membrane can be observed [1]. Exogenous fungal endophthalmitis often presents with a latency period of weeks to months after intraocular inoculation. Wykoff et al. reported that the timing of onset of symptoms varies from 24 h to 6 months after the injury, with an average of 1.8 months in cases of open-globe injury [2]. There are some reasons for delayed diagnosis of fungal infections. Fungal isolates are often regarded as contamination and it usually takes 4–6 weeks to detect slow-growing or fastidious organisms in fungal culture [3]. In addition, fungal endophthalmitis typically presents with subacute symptoms that worsen gradually [4]. Here, we report a case of lenticular infection caused by *Aspergillus* diagnosed 13 weeks after traumatic corneal laceration.

## Case presentation

**Fig. 1:**
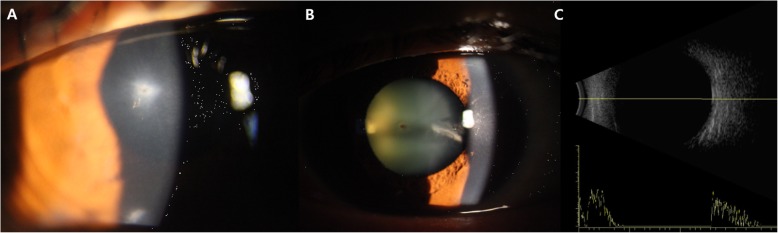
Anterior-segment photographs and B-scan sonography image at initial clinical presentation. **a**: Central corneal full-thickness laceration and little anterior chamber reaction at initial presentation. **b**: Anterior lens capsular rupture with a suspected foreign body. **c**: No evidence of intraocular foreign bodies or vitreous opacities on B-scan sonography

**Fig. 2:**
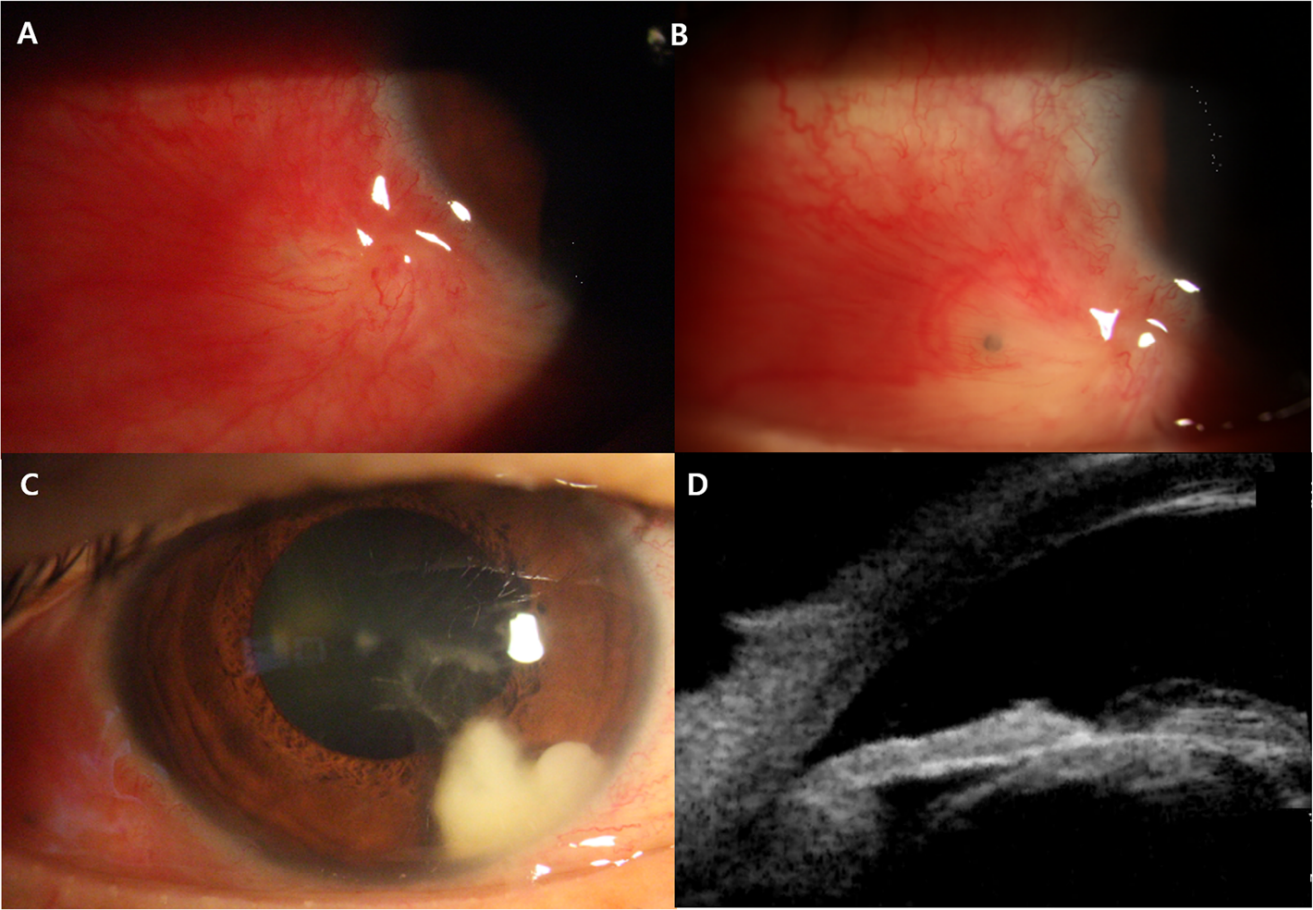
Anterior-segment photographs and ultrasound biomicroscopy (UBM) image. **a**: Focal nodular conjunctival injection persisting 10 days after the trauma, requiring topical steroid administration. **b**: Detection and surgical removal of the subconjunctival foreign body 6 weeks after the trauma. **c**: Worsening of the conjunctival injection and anterior chamber reaction and formation of thick fibrotic membrane on the anterior lens capsule around the traumatic lens opacity with white fluffy exudative material, suspected as a fungal ball, 10 weeks after the trauma. **d**: UBM image in 6 o’clock position of the anterior chamber angle showing highly reflective exudates, suspected as fibrotic membrane, on both the anterior and posterior sides of the anterior lens capsule

A 60-year-old woman with no history of systemic diseases presented with traumatic corneal laceration after being hit with a chestnut. Best-corrected visual acuity in the right eye was 0.4 (decimal value), and the intraocular pressure (IOP) was 19 mmHg. On slit-lamp examination, conjunctival injection, full thickness corneal laceration and anterior chamber reaction were found with positive Siedel’s test. In dilated eye examination, focal rupture of the anterior capsular bag and mild lens opacity were observed, but any visible foreign body was not detected in lens. There was no radiological evidence of intraocular foreign bodies on computed tomography (CT) or B-scan (Fig. 1). Under the precise slit-lamp examination, no chestnut thorn was found in conjunctiva or cornea. A bandage contact lens was inserted, and treatment was commenced with both topical (moxifloxacin 5 mg/mL, fortified tobramycin 14 mg/mL) and broad-spectrum systemic antibiotics. Ten days after the trauma, the corneal wound was sealed, and there was no active anterior chamber inflammation, but the focal nodular conjunctival injection persisted (Fig. 2-a). There was a possibility of accompanied scleritis; therefore, a topical steroid (prednisolone acetate 10 mg/mL) was prescribed. In the 6-week follow-up, subconjunctival foreign body (Fig. 2-b) was detected and surgically removed after conjunctival injection and edema being subsided (1st surgery). However, the conjunctival injection and anterior chamber reaction kept worsening.

In the 10-week follow-up, formation of a thick fibrotic membrane on the anterior lens capsule around the traumatic lens opacity and exudative material in the anterior chamber, suspected as a fungal ball, were observed abruptly (Fig. 2-c). Diagnostic surgery including scleral exploration and anterior chamber paracentesis was planned because there was a possibility of fungal infection by vegetable matter or remnant intralenticular foreign body. As the foreign body was organic chestnut thorn, and the white fluffy exudative material in the anterior chamber suggested a fungal infection, intracameral and subconjunctival amphotericin B injections(0.01 mg/0.1 cc) were administered. The remnant scleral and anterior capsular foreign bodies were surgically removed, and the specimen was sent to the department of laboratory medicine for KOH smear and culture tests. (2nd surgery). MacConkey agar, blood agar plate (BAP), chocolate agar plate, and SDA plates were incubated for 16 h (overnight) at 37 °C in bacteriological incubator. Once colonies were grown on the plate media, they were gram stained and individual cells were observed under a microscope. Then we asked outside professional facility to perform an antimicrobial susceptibility test and identify the organism. After the second surgery, the patient was prescribed 1% voriconazole eye drops and systemic fluconazole but without the topical steroid as a fungal infection was highly suspected from the clinical presentation. The KOH smear test of scleral foreign body was negative for fungal hyphae. In contrast, unspecified mold form species were grown from SDA plate.

**Fig. 3:**
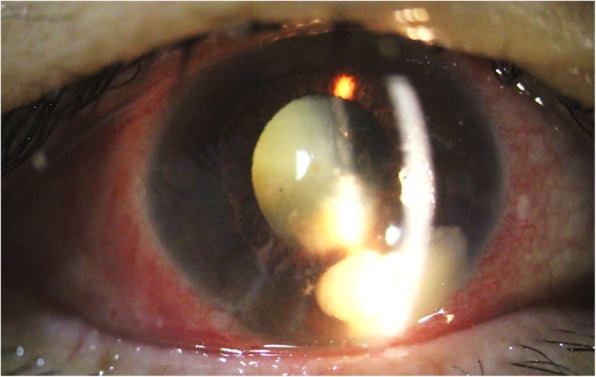
Anterior-segment photograph. Conjunctival injection persisting after surgical removal of remnant foreign bodies from the sclera, anterior capsule, and cornea. Enlarged hypopyon, suspected as a fungal ball, and worsened whitish plaque on the anterior lens surface

One week after the 2nd surgery, hypopyon and whitish plaque on the anterior lens surface progressed (Fig. 2-c, d). Therefore, intracameral amphotericin B injection (0.01 mg/0.1 cc) and removal of the suspicious remnant corneal foreign body were performed (3rd surgery). Although antifungal drugs, including antibiotics, were administered continuously, the exudative material in the anterior chamber gradually increased in size (Fig. 3).

Twelve weeks after the trauma, phacoemulsification through scleral tunnel incision (2.8 mm) without IOL insertion, repeated KOH smear and culture test of the anterior lens capsule, and intracameral amphotericin B injection (0.01 mg/0.1 cc) were performed (4th surgery). Fungal hyphae was detected on KOH smear and Aspergillus was grown on SDA plate from both anterior capsule and fibrotic membrane after the cataract surgery. Aspergillus infection was diagnosed 13 weeks after the trauma and 5% natamycin eye drop was prescribed additionally.

**Fig. 4:**
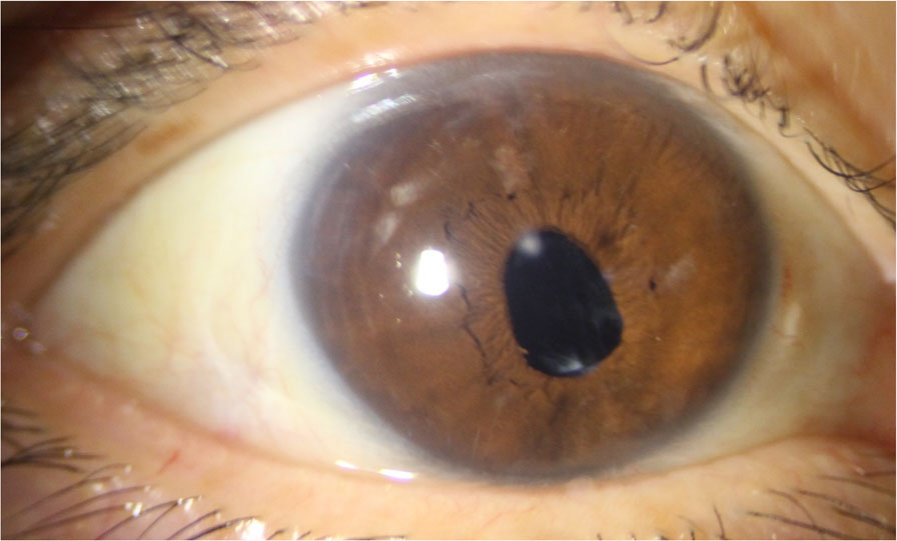
Anterior-segment photograph. Secondary insertion of 3-piece hydrophobic intraocular lens in the ciliary sulcus 27 weeks after the trauma, leading to a postoperative best-corrected visual acuity of 0.9 (decimal value) in the right eye and a small corneal opacity without conjunctiva or anterior chamber inflammation

**Fig. 5:**
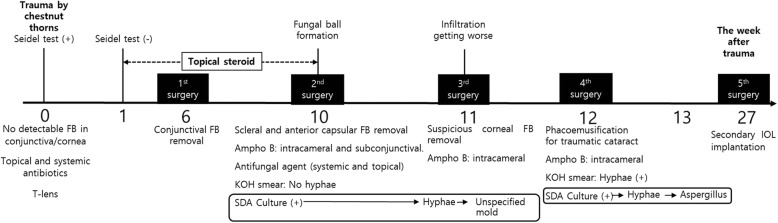
Disease course after the trauma. At first, there was no visible foreign body in ocular tissue therefore therapeutic contact lens was inserted for treatment of corneal perforation. A week later, corneal wound was sealed spontaneously, and topical steroid was prescribed to control the inflammation of conjunctiva and sclera. Six weeks after the trauma, the conjunctival foreign body was surgically removed (1st surgery). Ten weeks after the trauma, a fungal ball was observed in the anterior chamber. Scleral and anterior lens capsular foreign bodies were surgically removed (2nd surgery). Simultaneous intracameral and subconjunctival amphotericin B injections (0.01 mg/0.1 cc) were administered. Eleven weeks after the trauma, the suspected corneal foreign body was removed, and intracameral amphotericin B (0.01 mg/0.1 cc) was injected again (3rd surgery). A culture test of the scleral foreign body showed fungal hyphae, and an unspecified mold form was subsequently cultured. Twelve weeks after the trauma, phacoemulsification for traumatic cataract extraction and intracameral amphotericin B administration were performed (4th surgery). A KOH smear test of anterior capsule and fibrotic membrane obtained from 4th surgery showed fungal hyphae. Thirteen weeks after the trauma, *Aspergillus* was isolated in the fungus culture. Twenty-seven weeks after the trauma, a secondary intraocular lens was inserted (5th surgery)

After the 4th surgery, the anterior chamber inflammation improved. Three months after the phacoemulsification, the patient underwent a secondary insertion of 3-piece hydrophobic intraocular lens (IOL) in the ciliary sulcus (5th surgery), and her postoperative best-corrected visual acuity in the right eye was 0.9 in decimal visual acuity values (Fig. 4). The timeline for the disease progress and treatment is presented in Fig. 5.

## Discussion and conclusion

At initial clinical presentation, the patient was diagnosed with traumatic cataract, small anterior capsular tear, and inflammation of the conjunctiva and cornea, however embedded foreign bodies were not found in thorough slit-lam and dilated eye exam. They were detected over time and removed sequentially in four surgical procedures. Traumatic cataract developed slowly in the follow-up period, so cataract surgery was not considered in the absence of lens opacity progression. Tiny chestnut thorns affected the anterior segment including the cornea, conjunctiva, sclera, and crystalline lens simultaneously and made the diagnosis and treatment difficult. The extent of the damage should be evaluated precisely, as multiple tissues can be injured by thorns simultaneously. Tiny foreign bodies, such as parts of thorns, can be detected a few months after the trauma. Topical steroids were prescribed as the conjunctival nodular injection was not improved even after the removal of subconjunctival foreign body.

Anterior chamber inflammation suddenly aggravated, accompanied with whitish exudation formation, like a fungal ball, adjacent to the anterior lens capsule. Formation of the whitish exudation strongly suggested a fungal infection. Exogenous fungal endophthalmitis usually has a latency period, lasting weeks to months, unlike bacterial endophthalmitis, which usually presents in days [5]. In this case, a fungal mass appeared in the anterior chamber 10 weeks after the trauma.

Topical corticosteroids administration to treat scleral and conjunctival inflammation delayed clinical presentation and diagnosis of lenticular fungal infection. In addition, a steroid could suppress the host’s immune response to the pathogen, so the fungus could penetrate the deeper tissues aggressively, leading to abrupt inflammation of anterior segment. Furthermore, fungi replicate more freely in the presence of corticosteroids due to suppression of the host’s inflammatory response [2, 6, 7]. In this case, the patient was 60 years old and had no systemic diseases. Inflammation was localized in the anterior segment, as the patient’s immune system was functional despite steroid administration.

Clinical features of fungal endophthalmitis include localization of infiltration in the anterior chamber, pupillary space, and anterior vitreous. When inflammation extends to the vitreous cavity, the treatment is difficult, and visual prognosis is generally poor [5]. In this case, the infection was localized in the anterior segment, and the final visual outcome was good despite delay in the diagnosis and treatment of fungal infection. The final best-corrected visual acuity was 0.9 (decimal value), and there are two possible reasons for the favorable visual prognosis. First, the small corneal perforation was sealed in a few days relatively quickly, as chestnut thorns are thin and have a sharp margin. Furthermore, the corneal opacity was small and localized, so the transparency and contour of the cornea were maintained. Second, the infection was localized in the anterior segment and did not extend to the posterior segment. Furthermore, the posterior lens capsule was intact. Despite remnant foreign bodies, the inflammatory reaction was controlled with antibiotics, corticosteroids, and the patient’s immune system. As the patient was immune-competent, the mechanisms of ocular immune privilege in the anterior segment worked effectively, consisting of the blood–retina barrier, immunosuppressive factors in the eye, and systemic immune responses such as anterior chamber-associated immune deviation (ACAID) [8].

Rajaraman R et al. reported that prompt surgical evacuation of lenticular abscess and extracapsular cataract extraction with systemic and local antimicrobial treatment were effective in 17 cases of traumatic lenticular abscess [9]. We removed the lenticular fungal abscess by phacoemulsification as gently as possible to preserve the lens capsule. Secondary IOL implantation was performed after the inflammatory response was controlled, as in the previous study. Good visual outcome (0.9, decimal values) was obtained with continued antifungal treatment. Some physicians prefer simultaneous IOL implantation in primary cataract extraction with or without the presence of intraocular foreign bodies; however, in most reported cases of simultaneous surgery, injuries were self-healing or were localized in the cornea [10, 11]. To date, there is no consensus on whether IOL implantation should be performed at the time of lens removal or later as an additional surgery. If IOL is implanted at the time of repair, risks of infection, inflammation, and IOP elevation may increase. However, primary IOL implantation is relatively effective with shortening of the visual rehabilitation time and reduction in the number of surgeries. Secondary IOL implantation can reduce the potential risk of infection, but it can be a complicated procedure, including separation of synechiae among the lens capsule, iris, and cornea. Furthermore, delayed visual recovery can affect binocular function, causing ocular discomfort [10–12]. In this case, secondary IOL implantation was performed, as the primary goal of cataract extraction was removal of the infection source, and the inflammatory synechiae below the lens capsule was severe. After inflammation subsided, secondary IOL implantation was planned. Compared to hydrophilic IOL, 3-piece hydrophobic IOL is stable for an inflammatory condition. Moreover, long haptics are beneficial for IOL centration and refractive stability. Therefore, hydrophobic, monofocal 3-piece acrylic IOL was chosen.

Post-traumatic primary intralenticular fungal infections are rare. In many cases of lenticular infection, the lens is secondarily affected by the infection spreading from an anterior or posterior-chamber fungal mass [1]. However, in this case, the penetrating injury involved the lens directly. When patients present with trauma in the anterior segment of the eyeball from sharp-edged fine thorns, direct fungal inoculation of the lens should be suspected. Furthermore, fungal infections after an open-globe injury are rare but should be primarily considered, especially in cases of trauma from foreign matter such as thorns, branches, stones, and mud particles [5, 13]. Therefore, a prescription of topical corticosteroids to control the inflammatory reaction should be considered carefully, and thorough inspection is essential before steroid administration.

Multiple simultaneous injuries from tiny organic foreign matter increase the risk of fungal infection, and in cases of inflammatory reaction, a delayed fungal infection should be suspected. As this case shows, delayed fungal intralenticular infection in immunocompetent patients with localization in the anterior segment may have a good visual prognosis.

## Data Availability

All the data supporting our findings are contained within the manuscript.

## Abbreviations

SDA: Sabouraud dextrose agar
IOP: Intraocular pressure
CT: Computed tomography
BAP: Blood agar plate
IOL: Intraocular lens
ACAID: Anterior chamber-associated immune deviation
UBM: Ultrasound biomicroscopy
